# Os Supranaviculare as a Predisposing Factor to Navicular Stress Fractures

**DOI:** 10.5334/jbsr.2666

**Published:** 2021-11-19

**Authors:** Doaa Azawi, Amin Mahsouli, Filip Vanhoenacker

**Affiliations:** 1 AZ Sint-Maarten Mechelen, BE; 2 UCLouvain, BE; 3 AZ Sint-Maarten and University (Hospital) Antwerp/Ghent, BE

**Keywords:** Os Supranaviculare, Navicular Bone Stress Fracture, Radiography, Computed Tomography, Magnetic Resonance imaging

## Abstract

**Teaching Point:** An os supranaviculare is a variant of the anatomy which however may predispose to symptoms.

## Case presentation

**Figure 1:**
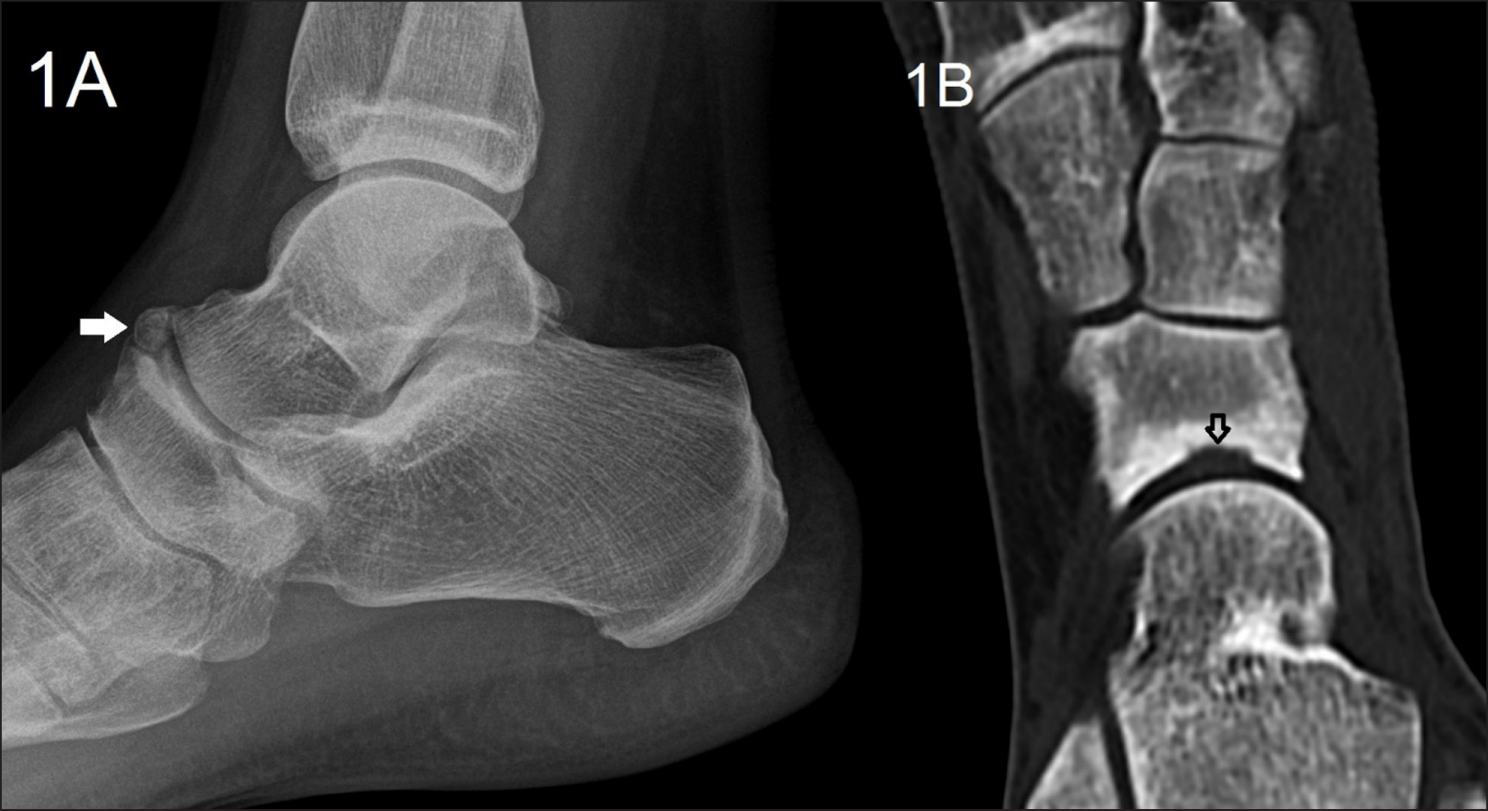


**Figure 2:**
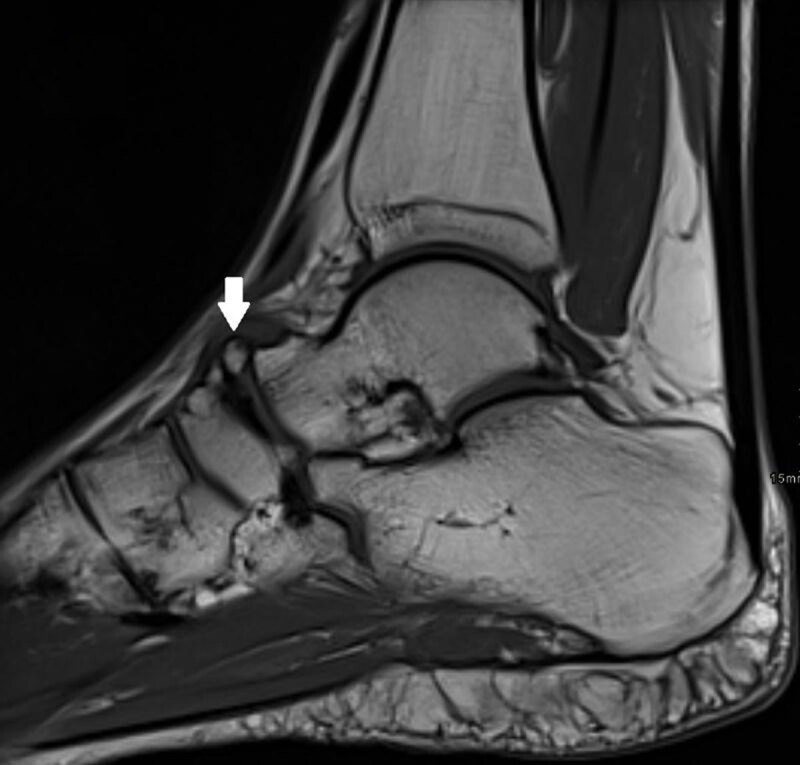


**Figure 3:**
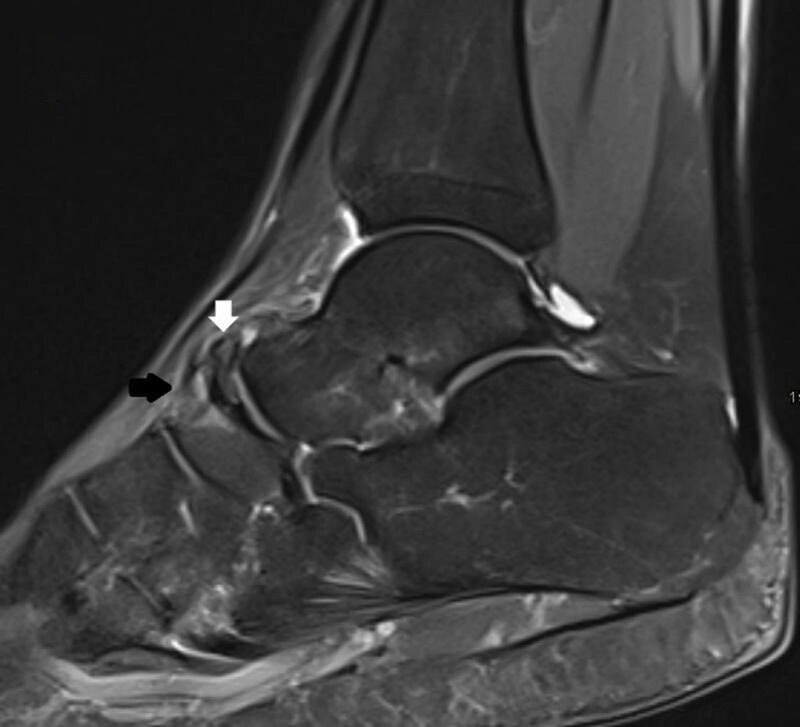


A 36-year-old athletic female presented with persistent pain at the dorsal side of the left ankle particularly with plantarflexion of the foot for eleven months. The pain aggravated following an ankle distortion. Conventional radiography showed an os supranaviculare (***Figure 1A***, arrow). There was sclerotic delineation of the articular surface of the navicular bone. Computed Tomography (CT) revealed a small sclerotic delineated cortical notch at the dorsal aspect of the proximal articular surface of the navicular bone (***Figure 1B***, oblique axial image, open arrow). Subsequent Magnetic Resonance Imaging (MRI) confirmed the os supranaviculare on T1-weighted images (WI) (***Figure 2***, arrow). Sagittal fat suppressed (FS) T2-weighted images revealed bone marrow edema in the os supranaviculare (***Figure 3***, white arrow) and in the dorsal aspect of the adjacent navicular bone (***Figure 3***, black arrow).

The diagnosis of a symptomatic os supranaviculare with stress reaction at the adjacent navicular bone was made.

## Comment

The os supranaviculare is an accessory ossicle located at the proximal dorsal aspect of the navicular bone or talonavicular joint. It is present in approximately 1% of the population.

Though considered a normal variant in most scenarios, it may become symptomatic due to the occurrence of navicular stress fractures. The exact cause of this association is unclear, but a pre-existing dorsal cortical notch accompanying an os supranaviculare, like in our case, may contribute to concentrating bone stress on the dorsal aspect of the navicular bone [1].

Tarsal navicular stress fractures are more commonly observed in athletes involved in sprinting and jumping sports. Contrary to what was previously suspected, hypovascularity of the central, dorsal and proximal part of the navicular bone occurs in a relatively small proportion of individuals, while biomechanical and other clinical factors may play a more prominent role in developing navicular stress fractures. Other mechanical risk factors for developing navicular stress fractures include pes cavus, metatarsus adductus, limited subtalar or ankle motion, medial narrowing of the talonavicular joint, as well as a short first metatarsal.

Radiographs may show the supernumerary bone but have very low sensitivity for navicular stress fractures. MRI remains the examination of choice by demonstrating bone marrow edema.

In our patient, bone marrow edema is clearly visible on FS T2-WI but barely subtle on T1-WI, in keeping with a stress reaction grade 3.

In conclusion, the presence of a prominent os supranaviculare and pain at the dorsal aspect of the foot should alert the radiologist for the possibility of symptomatic os supranaviculare. MRI is the next step in the diagnostic process following conventional radiography for demonstrating bone marrow edema in case of symptomatic os supranaviculare.
